# Sphingosine-1-phosphate induced epithelial-mesenchymal transition of hepatocellular carcinoma via an MMP-7/syndecan-1/TGF-β autocrine loop

**DOI:** 10.18632/oncotarget.11450

**Published:** 2016-08-20

**Authors:** Ye Zeng, Xinghong Yao, Li Chen, Zhiping Yan, Jingxia Liu, Yingying Zhang, Tang Feng, Jiang Wu, Xiaoheng Liu

**Affiliations:** ^1^ Institute of Biomedical Engineering, School of Preclinical and Forensic Medicine, Sichuan University, Chengdu, China; ^2^ State Key Laboratory of Oncology in South China, Department of Radiation Oncology, Sun Yat-Sen University Cancer Center, Guangzhou, China

**Keywords:** sphingosine-1-phosphate, syndecan-1, TGF-β, epithelial-mesenchymal transition, hepatocellular carcinoma

## Abstract

Sphingosine-1-phosphate (S1P) induces epithelial–mesenchymal transition (EMT) in hepatocellular carcinoma (HCC). However, its underlying mechanism remains largely unknown. In the present study, we investigated the correlation between S1P and syndecan-1 in HCC, the molecular mechanism involved, as well as their roles in EMT of HCC. Results revealed a high serum S1P level presents in patients with HCC, which positively correlated with the serum syndecan-1 level. A significant inverse correlation existed between S1P_1_ and syndecan-1 in HCC tissues. S1P elicits activation of the PI3K/AKT signaling pathways via S1P_1_, which triggers HPSE, leading to increases in expression and activity of MMP-7 and leading to shedding and suppression of syndecan-1. The loss of syndecan-1 causes an increase in TGF-β1 production. The limited chronic increase in TGF-β1 can convert HCC cells into a mesenchymal phenotype via establishing an MMP-7/Syndecan-1/TGF-β autocrine loop. Finally, TGF-β1 and syndecan-1 are essential for S1P-induced epithelial to mesenchymal transition. Taken together, our study demonstrates that S1P induces advanced tumor phenotypes of HCC via establishing an MMP-7/syndecan-1/TGF-β1 autocrine loop, and implicates targetable S1P_1_-PI3K/AKT-HPSE-MMP-7 signaling axe in HCC metastasis.

## INTRODUCTION

Hepatocellular carcinoma (HCC) is a common and aggressive human malignancy. HCC is the third and the second cause of cancer-related death in the Asia-Pacific region and worldwide [1, 2]. Despite greater effort has been made to improve its diagnosis and treatment [3], the five-year survival of HCC is less than 50% [2]. Metastasis is the most deadly and least understood aspect of cancer, being in charge of the high mortality rate of HCC [4]. Metastasis is a multistep process. Although increasing evidence indicates that Epithelial–mesenchymal transition (EMT) is an initial and critical step involved in HCC tumor metastasis [5], the mechanism of EMT in HCC remains largely unknown.

Numerous studies demonstrated that tumor cell-microenvironment interactions regulate the process of EMT via regulation of the expression of growth factors, cytokines and matrix metalloproteinases (MMPs) [6, 7]. Transforming growth factor β1 (TGF-β1) plays a critical role in the induction of EMT [8]. Recent studies have shown that sphingosine-1-phosphate (S1P) is linked with the EMT of A549 via regulation of TGF-β1 autocrine, contributing to pulmonary fibrosis [9, 10]. S1P also modulated the levels of MMPs such as MMP-2 and MMP-9, regulating cell invasion [11–13]. Evidence has further shown that S1P and its receptors associated with the development and progression of HCC [14, 15]. Serum S1P was upregulated in the patients with HCC, compared with patients with cirrhosis [15]. S1P elicits cellular responses mainly via binding to a family of the G protein-coupled receptor [16]. The S1P receptor subtypes, including S1P_1_, S1P_2_, and S1P_3_ are ubiquitously expressed [17]. In general, S1P_1_ is exclusively coupled with G_i_ protein to activate cell migration through extracellular signal-regulated kinase, PI3K/Akt, phospholipase C and Rac signaling pathways, whereas the S1P_2_ and S1P_3_ receptors could couple with the G_i_, G_q_ and G_12/13_ proteins to inhibit cell migration via Rho/Rho kinase pathway [18]. It was demonstrated that the increase in S1P level promotes cell migration and invasion through the S1P_1_ in hepatocellular carcinoma cells [19]. Although S1P has been implicated in EMT, the molecular mechanism involved in HCC is still not clear.

We previously established a role of S1P in glycocalyx integrity via inhibiting shedding of syndecan-1 ectodomain with attached sulfated glycosaminoglycan (sGAG) including chondroitin sulfate and heparan sulfate (HS), and inducing syndecan-1 synthesis in endothelial cells [20, 21]. In HCC patients, the serum syndecan-1 exhibited a higher level than healthy individuals [22], and syndecan-1 expression was suppressed in HCC tissue than in non-tumoral liver tissue [23]. Therefore, we hypothesized that a correlation exists between the S1P and syndecan-1 in HCC, which might be critical molecules in the EMT of HCC.

Thus, in the present study, we investigated the effect of S1P on syndecan-1 in HCC. We also highlighted the S1P_1_-PI3K/AKT-heparanase (HPSE) -MMP-7 and S1P_1_/HPSE-ERK1/2 signaling axes in HCC, which is responsible for S1P-induced shedding and -inhibited synthesis of syndecan-1, leading to TGF-β1 production. The resulting TGF-β1 secretion and expression further enhanced MMP-7 activity, leading to syndecan-1 shedding and thus establishing an MMP-7/syndecan-1/TGF-β1 autocrine loop to convert HCC cells into a mesenchymal phenotype. Those might be targeted for developing novel strategies to decrease mortality and to improve the prognosis of HCC.

## RESULTS

### S1P and shed syndecan-1 in serum are enhanced in HCC patients

**Figure 1:**
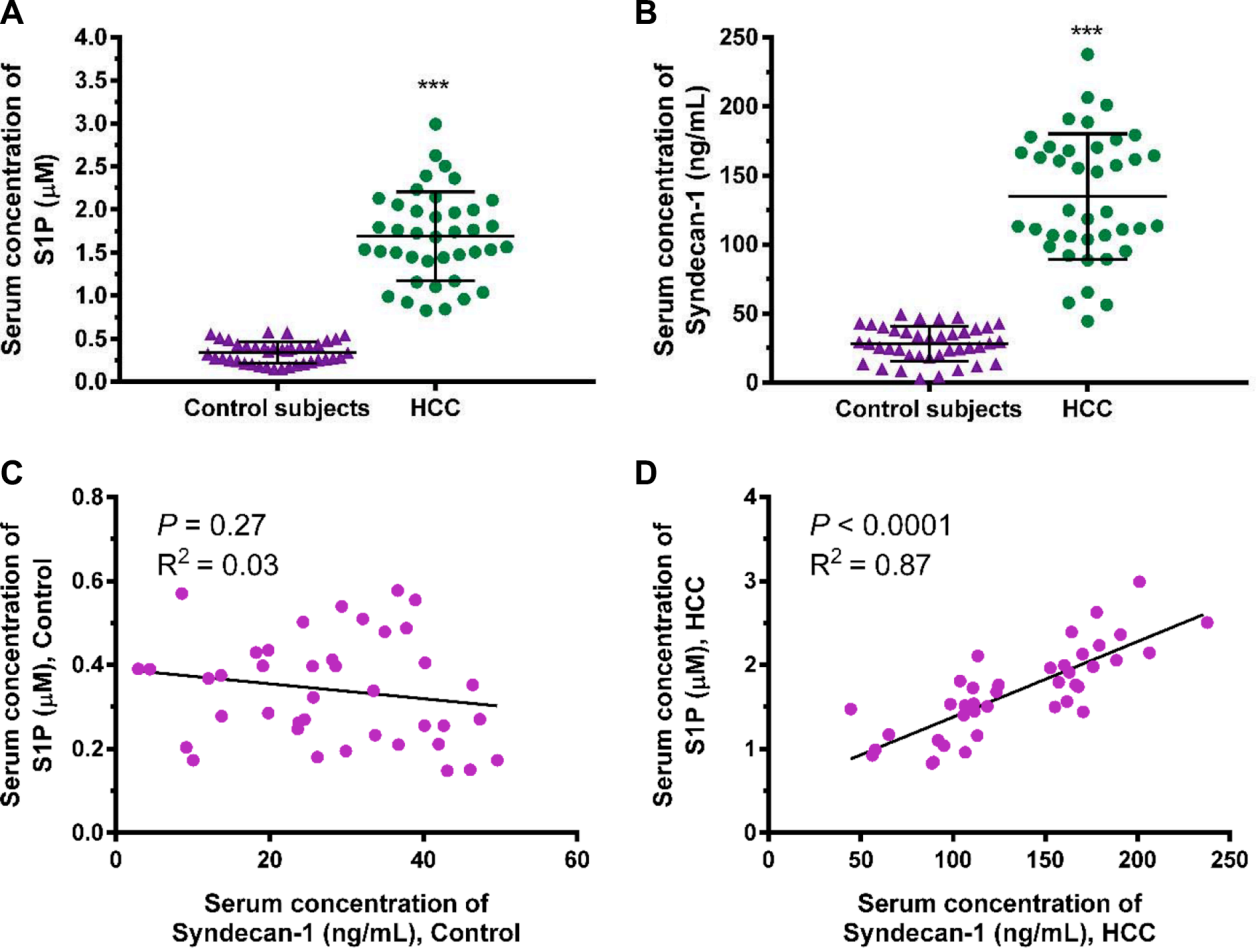
Serum sphingosine-1-phosphate (S1P) and syndecan-1 concentrations are increased in patients with hepatocellular carcinoma (HCC) compared with healthy controls (**A)** and (**B**), S1P (A) and syndecan-1 (B) concentrations were measured by S1P and syndecan-1 ELISA assays in serum from 40 healthy controls and 40 patients with HCC. The results are shown as the median of triplicate determinations for each sample. The lines indicate Mean ± S.D. Asterisks indicate significance at ***P < 0.001 (ANOVA). (**C**) and (**D**), the correlation between serum S1P and syndecan-1 in healthy controls (C) and HCC patients (D) were determined by Pearson analyses.

We investigated the correlation between S1P and shed syndecan-1 using the serums collected from 40 healthy controls and 40 HCC patients (Figure 1). The serum S1P and syndecan-1 levels were significantly higher in HCC than in healthy controls (Figure 1A and 1B). Further analysis revealed a positive correlation between S1P and shed syndecan-1 in HCC patients, but did not in healthy controls (Figure 1C and 1D). Thus, S1P positively correlated with shed syndecan-1 in HCC patients. The interaction between S1P and syndecan-1 reflects the progression of HCC.

### A significant inverse correlation exists between S1P_1_ and syndecan-1 in HCC patients

**Figure 2:**
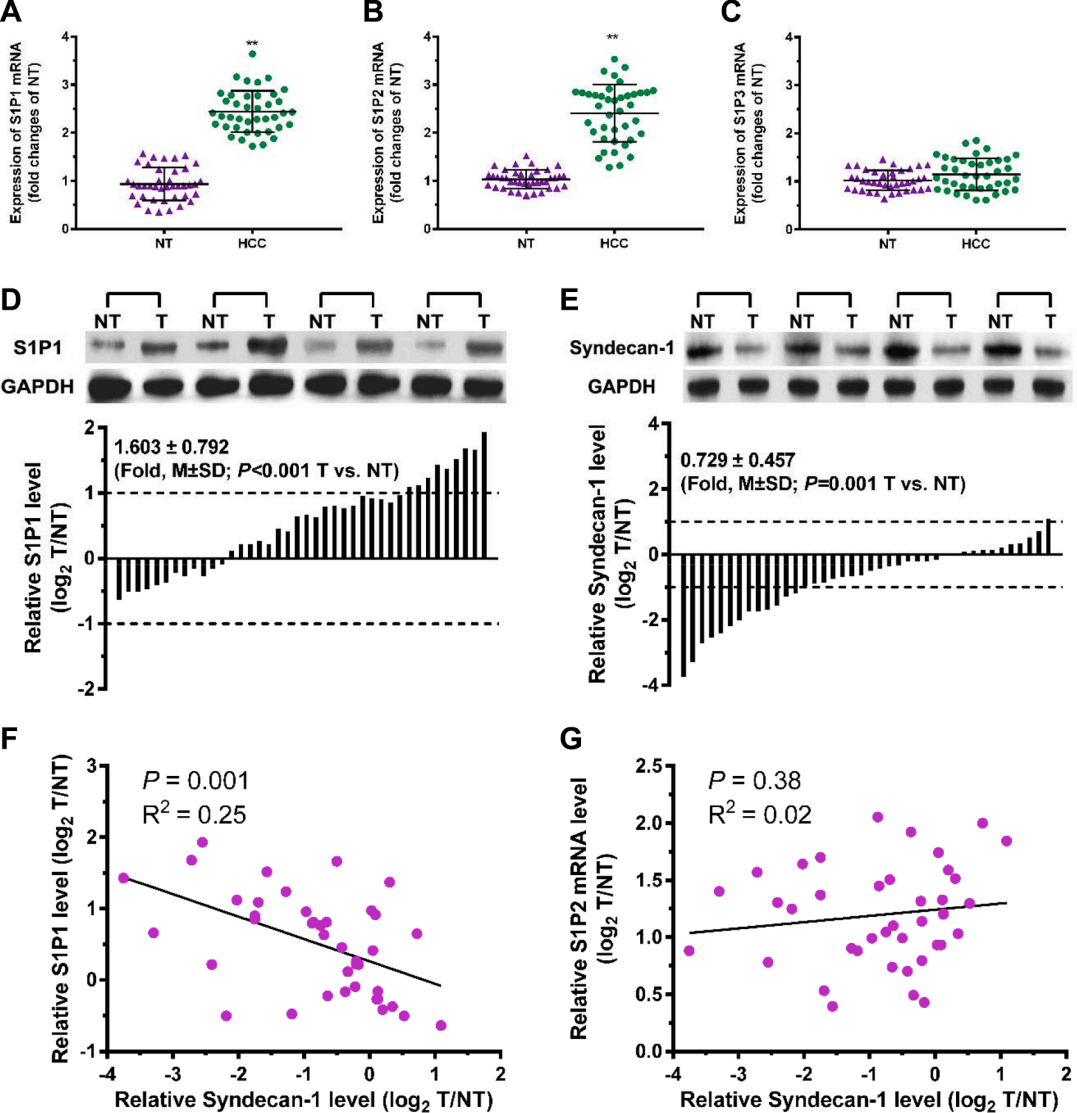
A correlation exists between S1P_1_ and syndecan-1 in HCC and para-carcinoma tissues (**A**), (**B**) and (**C**), mRNA expressions of S1P_1_ (A), S1P_2_ (B), and S1P_3_ (C) were measured by qRT-PCR assays in 40 pairs of HCC and their adjacent non-carcinoma tissues (NTs). The results are shown as the median of triplicate determinations for each sample. The lines indicate Mean ± S.D. Asterisks indicate significance at **P < 0.01 (ANOVA). (**D**) and (**E**), immunoblottings for S1P_1_ (D) and syndecan-1 (E). Protein levels were determined by Western blot in pairs of HCC (T) and NTs. If log_2_ fold change (log_2_ T/NT) more than 1 or less than -1, the expressions of S1P_1_ and syndecan-1 were considered overexpression or suppression, respectively. (**F**) and (**G**), correlations between S1P_1_ and syndecan-1 (F), and between S1P_2_ mRNA and syndecan-1 (G) were determined.

Forty pairs of HCC tumor and adjacent non-tumorous (NTs) tissues were collected to assess the relationship between S1P receptors and syndecan-1 in HCC (Figure 2). The mRNA expression levels of S1P_1_ and S1P_2_ in HCC tumor tissues were significantly upregulated, compared with NTs (Figure 2A and 2B). No significant difference was observed in the expression of S1P_3_ mRNA between HCC tissues and NTs (Figure 2C). In the immunoblottings, S1P_1_ levels were also upregulated to a great extent in HCC tumor tissues (Figure 2D). Among 40 patients with HCC, 28 cases (70%) exhibited a higher level of S1P_1_ and nine cases (22.5%) upregulated by at least two-fold as compared with the corresponding NTs. In contrast to the changes of syndecan-1 in serum, syndecan-1 was significantly downregulated in HCC tumor (Figure 2E), its expression in thirteen cases (32.5%) was approximately two-fold lower in HCC tumor, compared with corresponding NTs. In the HCC specimens, S1P_1_ inversely related to syndecan-1 (Figure 2F), whereas those of S1P_2_ mRNA did not (Figure 2G). Thus, a significant inverse correlation was demonstrated between S1P_1_ and syndecan-1 in HCC tumor.

### S1P_1_, HPSE, and MMPs play essential roles in inhibition and the shedding of syndecan-1 in the presence of S1P

**Figure 3:**
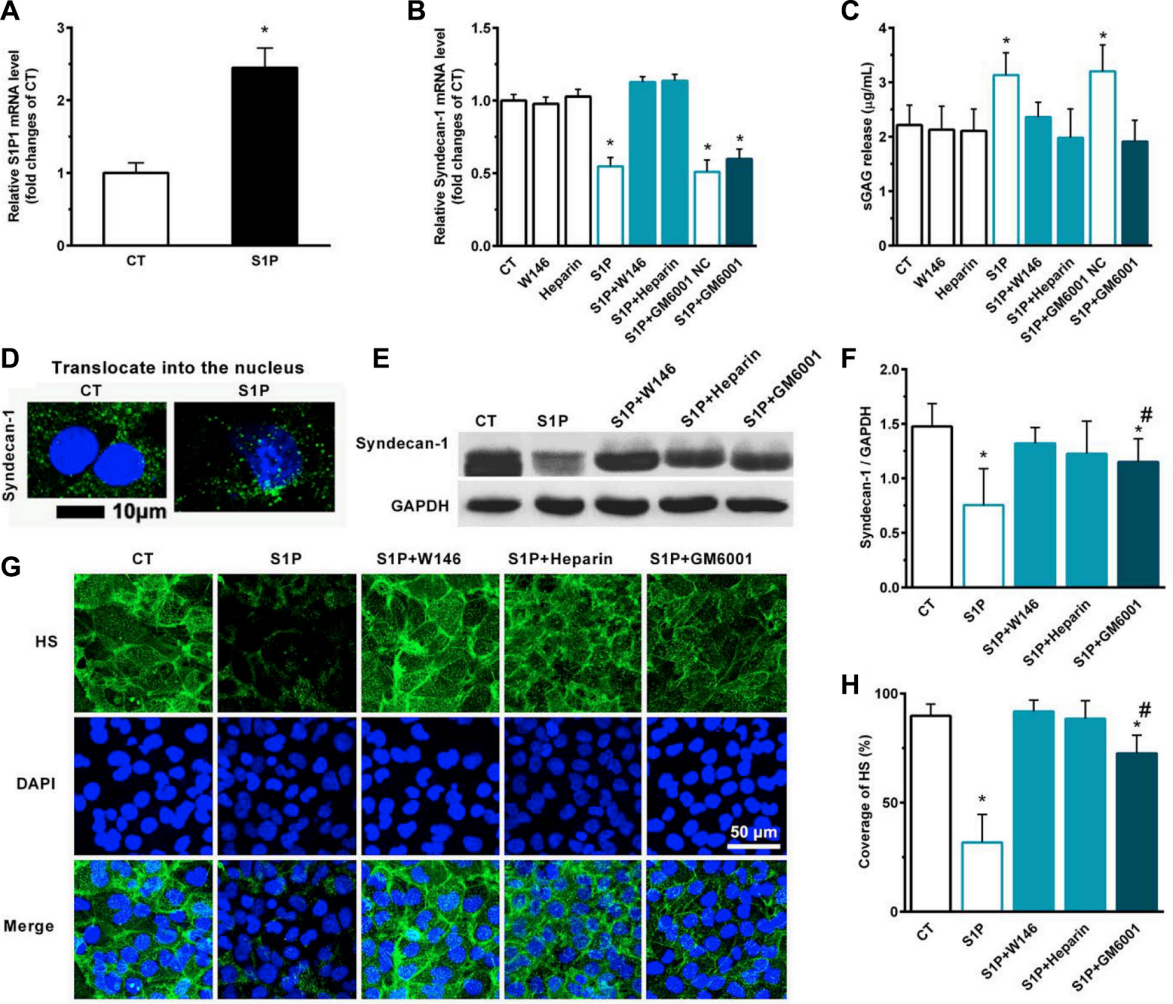
S1P-suppressed synthesis of syndecan-1 is mediated by S1P_1_, heparanase (HPSE), and S1P-induced shedding of syndecan-1 is mediated by S1P_1_, HPSE, and matrix metalloprotease (MMP) Human liver hepatocellular cells (HepG2) were stimulated in the presence or absence of 2 μM S1P for 72 h. (**A**) S1P increased mRNA expression of S1P_1_. HepG2 cells were treated with S1P_1_ inhibitor W146 (10 μM), HPSE inhibitor low molecular weight heparin (50 μg/ml), generic MMP inhibitor Ilomastat (GM6601, 10 μM), or GM6001 negative control (NC) for 30 min before S1P stimulation for 72 h. (**B**) S1P downregulated mRNA transcripts of syndecan-1, which was blocked by W146 and heparin, but was not by Ilomastat. (**C**) S1P-induced sGAG release from HepG2 cells was eliminated by W146, heparin, and Ilomastat, respectively. sGAG in the culture medium was detected by using 1,9-dimethylmethylene blue (DMMB) assay. (**D**) S1P decreased syndecan-1 staining (*green*) but induced translocation of syndecan-1 into the nucleus (DAPI, *blue*). Confocal images depict a *z*-plane through the center of the cell nucleus. Scale bar, 10 μm. (**E**) and (**F**), a representative image of Western blots (E) with densitometric quantification (F) of syndecan-1 in cell lysates after 30 min exposure of W146, heparin, and Ilomastat before S1P treatment for 72 h. Results are presented as a ratio to GAPDH. (**G**) and (**H**), immunofluorescence images (maximum-intensity projections of a confocal *z*-stack) (G) and the coverage of heparan sulfate (HS, *green*). Nuclei were counterstained with DAPI (*blue*) (H). *P < 0.05 as compared with CT; ^#^*P <* 0.05 as compared with S1P (ANOVA).

We detected the expression of syndecan-1 and the sulfate glycosaminoglycan (sGAG) level in media in the presence of S1P. S1P induced the expression of S1P_1_ mRNA in HepG2 cells (Figure 3A). S1P suppressed the expression of syndecan-1 mRNA (Figure 3B) and promoted the shedding of syndecan-1 (Figure 3C). Interestingly, S1P not only induced the inhibition and shedding of syndecan-1 but also stimulated the translocation of syndecan-1 into the nucleus (Figure 3D). S1P_1_ specific antagonist W146 and HPSE inhibitor heparin abolished the S1P-induced syndecan-1 mRNA expression, whereas generic MMP inhibitor Ilomastat (GM6001) did not (Figure 3B). W146, heparin, and GM6001 almost blocked the shedding of syndecan-1 (Figure 3C). Thus, S1P_1_ and HPSE involved in the expression and shedding of syndecan-1, whereas MMPs only affected the shedding of syndecan-1. Consistent with this, the expression of syndecan-1 in protein level was downregulated by S1P. This change was abrogated by W146 and heparin and was partially attenuated by GM6001 (Figure 3E and 3F). Moreover, a decrease in HS coverage was also observed in the presence of S1P. And this change was abrogated by W146 and heparin, or partially attenuated by GM6001 (Figure 3G, and 3H). Taken together, loss of syndecan-1 induced by S1P is due to its inhibition and shedding together. S1P suppressed the expression of syndecan-1 via S1P_1_ and HPSE while causing the shedding of syndecan-1 via S1P_1_, HPSE, and MMPs.

### HPSE induced by S1P further enhance expression and activity of MMP-7

**Figure 4:**
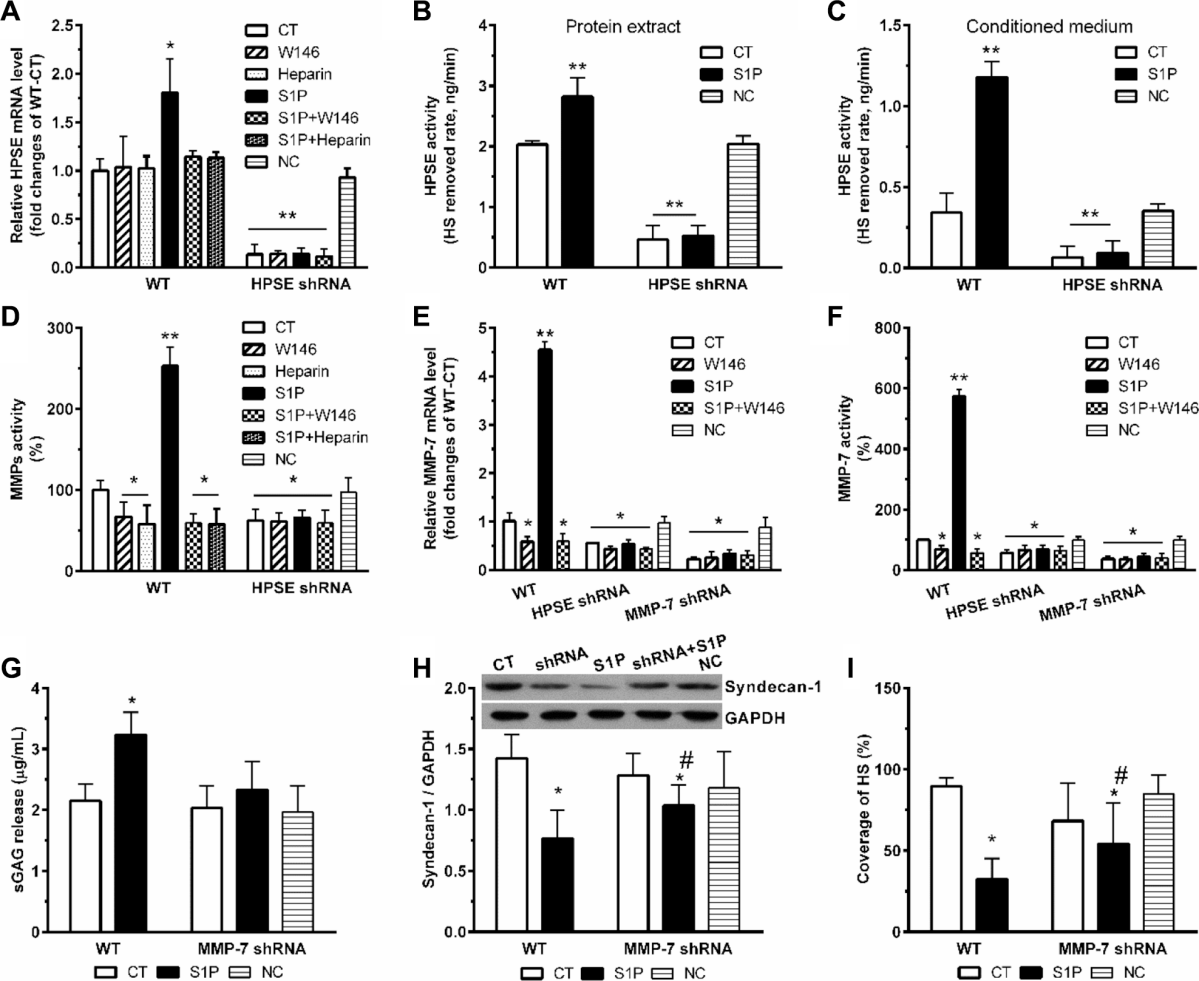
S1P-enhanced expression and activity of MMP-7 is dependent on HPSE HPSE knockdown (HPSE shRNA) and MMP-7 knockdown (MMP-7 shRNA) by shRNA transduction were performed. Then, cells were treated with S1P_1_ inhibitor W146 (10 μM), and HPSE inhibitor low molecular weight heparin (50 μg/ml) for 30 min before S1P stimulation for 72 h. Cells transfected with scrambled shRNA were set as a negative control (NC). HPSE expression and activity were modulated by S1P via S1P_1_. (**A**) HPSE mRNA expression levels in WT and HPSE shRNA cells were determined by qRT-PCR. The data were corrected to GAPDH as an internal control and represents the mean ± S.D. (Error bars) of at least three independent experiments performed in triplicate, and final results were normalized to the non-treated WT control cells. (**B**) and (**C**), HPSE activity in protein extracts (B) and conditioned media (C) in WT and HPSE shRNA cells. HPSE activity is expressed as HS removed rate, nanograms per minute. (**D**) MMPs activity in the culture medium was determined by using Sensolyte 390 generic MMP activity kit (AnaSpec). The results were normalized to the WT control cells. (**E**) MMP-7 mRNA expression levels in WT, HPSE shRNA, and MMP-7 shRNA cells. (**F**) MMP-7 activity in the culture medium was determined by using SensoLyte 520 MMP-7 assay kit (AnaSpec). MMP-7 shRNA and WT control cells were treated with 2 μM S1P for 72 h. Then, sGAG assay, syndecan-1 expression, and staining of HS were performed. (**G**) sGAG in the culture medium was measured by DMMB assay. (**H**) a representative image of Western blots with densitometric quantification of syndecan-1. (**I**), the coverage of heparan sulfate (HS) was estimated by using maximum-intensity projections of a confocal *z*-stack. *P < 0.05, **P < 0.01 as compared with CT; ^#^*P <* 0.05 as compared with S1P-treated WT control (ANOVA).

S1P induced the expression of HPSE mRNA, which was abolished by W146 and heparin (Figure 4A). The transcription level of HPSE reflected its activity. Significant increases in HPSE activity in both total cell lysates (Figure 4B) and culture media (Figure 4C) were also detected. As expected, transcription and activity of HPSE dramatically suppressed in HPSE-shRNA cells, and these were no longer activated by S1P (Figure 4A–4C). Similarly, W146 and heparin blocked the S1P-induced MMPs activity (Figure 4D). Importantly, S1P also no longer stimulated activation of MMPs in HPSE-shRNA cells, thus suggesting that MMPs activated by S1P was mediated by S1P_1_ and downstream HPSE activation.

To further figure out which MMP responsible for the shedding of syndecan-1, we examined the transcription level and activity of MMP-7. As predicted, S1P strikingly enhanced the transcription level (Figure 4E) and activity (Figure 4F) of MMP-7. Those changes were significantly abrogated by both HPSE knockdown and MMP-7 knockdown. In addition, MMP-7 knockdown abolished the changes in sGAG release (Figure 4G). Sole MMP-7 knockdown was insufficient to extinguish all the deregulation of syndecan-1 (Figure 4H) and loss of HS (Figure 4I). Therefore, inhibition of MMP-7 only protects syndecan-1 from S1P-induced shedding, stressing a vital role of MMP-7 in S1P-induced syndecan-1 shedding.

### S1P exerts its roles in suppression and shedding of syndecan-1 via activation of the PI3K/AKT and ERK1/2 pathways

**Figure 5:**
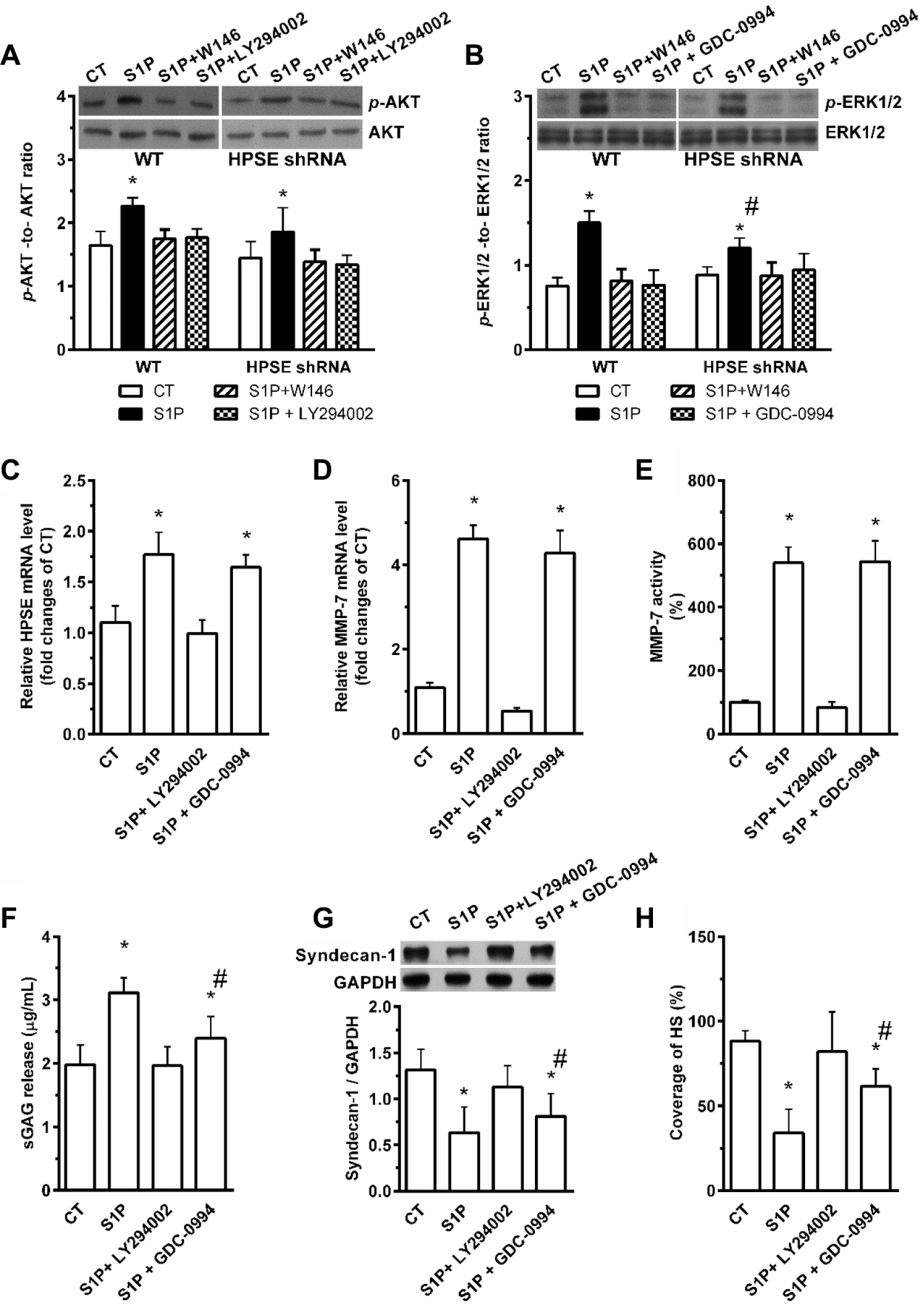
Involvement of PI3K/AKT and ERK1/2 pathways in S1P- S1P_1_-induced shedding and -suppressed the synthesis of syndecan-1 HepG2 WT and HPSE shRNA cells were stimulated with 2 μM S1P for 72 h with or without pretreatment for 30 min with S1P_1_ inhibitor W146 (10 μM), PI3K/AKT inhibitor LY294002 (20 μM) or ERK1/2 inhibitor GDC-0994 (50 μM). (**A**) and (**B**), the phosphorylation levels of AKT (A) and ERK1/2 (B) in WT and HPSE shRNA cells were evaluated by Western blot. Histograms show densitometric quantification of *p*-AKT or *p*-ERK normalized to total AKT or ERK, respectively. (**C**) LY294002 inhibited the S1P-induced expression of HPSE mRNA, but GDC-0994 did not. (**D**) and (**E**) LY294002 abolished the S1P-induced mRNA expression and activity of MMP-7, but GDC-0994 did not. The mRNA expression of MMP-7 in cell lysates was performed by qRT-PCR, and the activity of MMP-7 in cell mediums was determined by using SensoLyte 520 MMP-7 assay kit (AnaSpec). (**F**) S1P-induced sGAG release was blocked by LY294002 or in partly inhibited by GDC-0994. sGAG in culture medium was detected by DMMB assay. (**G**) and (**H**), effects of S1P in the suppression of syndecan-1 expression (G) and decrease in the coverage of HS (H) were blocked by LY294002 or in partly inhibited by GDC-0994. Syndecan-1 expression was measured by Western blot. The coverage of heparan sulfate (HS) was estimated by using maximum-intensity projections of a confocal *z*-stack. *P < 0.05 as compared with WT control cells; ^#^*P <* 0.05 as compared with S1P-treated WT control (ANOVA).

PI3K/AKT inhibitor LY294002 and ERK1/2 inhibitor GDC-0994 markedly blocked the S1P-induced the AKT (Figure 5A) and ERK1/2 (Figure 5B) phosphorylation, respectively. Knockdown of HPSE also decreased the S1P-induced phosphorylation level of ERK1/2 at least in part (Figure 5B). LY294002 suppressed S1P-induced transcription levels of HPSE (Figure 5C) and MMP-7 (Figure 5D), as well as the MMP-7 activity (Figure 5E), but GDC-0994 was without effect. It was suggested that HPSE and MMP-7 downstream to PI3K/AKT signaling pathway, and HPSE upstream to ERK1/2 signaling pathway.

The S1P-induced increase in sGAG release (Figure 5F) and decreases in syndecan-1 (Figure 5G) with attached HS (Figure 5H) were blocked by LY294002. However, the changes in levels of sGAG release, syndecan-1, and HS-induced by S1P were only partially abrogated by GDC-0994, implying a higher synthesis level of syndecan-1 will elevate its shedding level (data not shown). This further supports the PI3K/AKT signaling pathway mediated the shedding of syndecan-1 under S1P stimulation, and the ERK1/2 signaling pathway mediated the suppression of syndecan-1.

### S1P increases expression and secretion of TGF-β1, which, in turn, brings on the inhibition and shedding of syndecan-1

**Figure 6:**
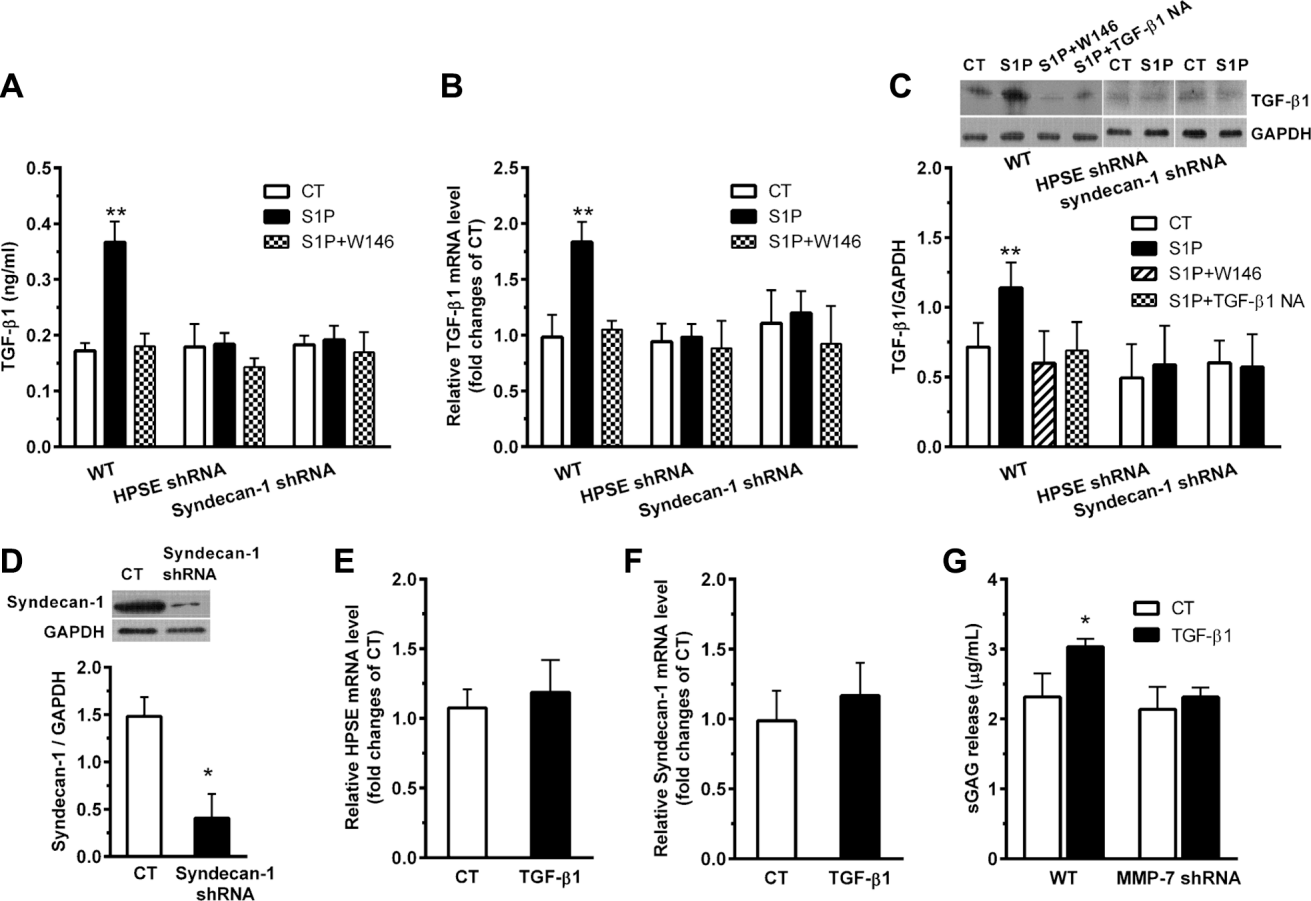
S1P induces expression and secretion of transforming growth factor-β1 (TGF-β1), which, in turn, enhances the suppression and shedding of syndecan-1 HepG2 WT, HPSE shRNA, and syndecan-1 shRNA cells were treated with 2 μM S1P for 72 h with or without pretreatment for 30 min with S1P_1_ inhibitor W146 (10 μM), ERK1/2 inhibitor GDC-0994 (50 μM), or 5 μg/ml neutralizing TGF-β1 antibody as indicated. (**A**), (**B**), and (**C**), S1P promoted TGF-β1 secretion in culture medium (A), and mRNA (B) and protein expression (C) as measured by ELISA, qRT-PCR and Western blot assay, respectively. Representative image of Western blots with densitometric quantification are shown for TGF-β1, and the results represent the mean ± S.D. as a ratio to GAPDH. (**D**) the knockdown of syndecan-1 by shRNA transduction was checked. HepG2 cells were stimulated with 0.5 ng/ml exogenous TGF-β1 for 72 h. (**E**) change in expression of HPSE mRNA in cells treated with TGF-β1 was not detected. HepG2 cells were pretreated with ERK1/2 inhibitor GDC-0994 (50 μM) for 30 min before exposure to 0.5 ng/ml exogenous TGF-β1 for 72 h. (**F**) TGF-β1 not significantly changed syndecan-1 mRNA expression. HepG2 WT and MMP-7 shRNA cells were stimulated with 0.5 ng/ml exogenous TGF-β1 for 72 h. (**G**) knockdown of MMP-7 abolished the TGF-β1-induced sGAG release. *P < 0.05, **P < 0.01 as compared with WT control cells; ^#^*P <* 0.05 as compared with S1P-treated WT control (ANOVA).

S1P increased TGF-β1 secretion (Figure 6A). S1P also upregulated TGF-β1 in both mRNA (Figure 6B) and protein (Figure 6C) levels. Those changes were diminished by W146, HPSE shRNA, and syndecan-1 shRNA, respectively. The knockdown of syndecan-1 was confirmed by demonstrating a very low expression level of syndecan-1 in syndecan-1 shRNA cells (Figure 6D). Moreover, the neutralizing TGF-β1 antibody was sufficient to inhibit the S1P-induced expression of TGF-β1 (Figure 6C), which upheld the restraint ability of this antibody on the S1P-induced production of TGF-β1.

Interestingly, despite TGF-β1 not influenced the expression of HPSE (Figure 6E) and syndecan-1 (Figure 6F), it had enhanced the level of sGAG release (Figure 6G). The elevated TGF-β1 level is tightly regulated by the MMP-7 (Figure 6G) that mediated the shedding of syndecan-1 (Figure 4), forming an MMP-7/syndecan-1/TGF-β1 autocrine loop.

### S1P-induced EMT indubitably requires syndecan-1 and TGF-β1

**Figure 7:**
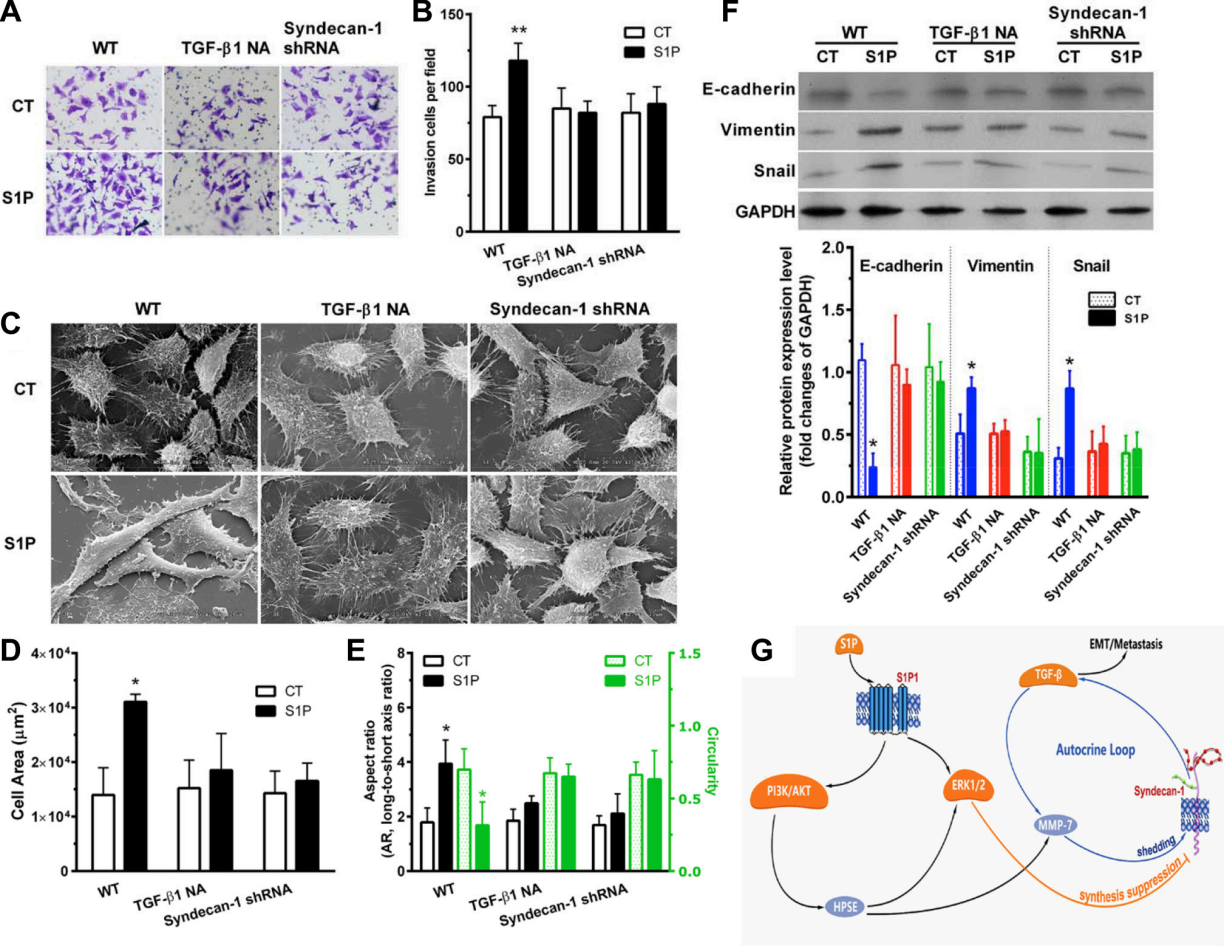
S1P-induced epithelial to mesenchymal transition (EMT) was mediated by syndecan-1 and TGF-β1 HepG2 control and syndecan-1 shRNA cells were treated with 2 μM S1P for 72 h with or without an addition of 5 μg/ml neutralizing TGF-β1 antibody. (**A**) and (**B**), HepG2 cells knocked down for syndecan-1 (syndecan-1 shRNA) compared with control cells were plated to invade through Matrigel-coated Transwell. An addition of 5 μg/ml neutralizing TGF-β1 antibody abolished S1P-induced cell invasion. The invaded cells were quantified and representative images are present. (**C**), (**D**), and (**E**), representative images were photographed under a scanning electron microscope (C). Alterations in cell area (D) and morphology (the aspect ratio and circularity, E) were evaluated by using image J. The results represent the mean ± S.D. of 80 cells from at least three images. (**F**) expression of epithelial phenotype marker E-cadherin and mesenchymal phenotype marker Vimentin, as well as transcription regulatory protein Snail, were analyzed by Western blotting with densitometric quantification. (**G**) a schematic summary of S1P-induced EMT via an MMP-7/syndecan-1/TGF-β1 autocrine loop. This pathway involves activation of PI3K/AKT signaling pathways via S1P_1_, which triggers an enzyme HPSE, leading to an increase in expression and activity of MMP-7 and leading to activation of the ERK1/2 signaling pathway. MMP-7 mediated the shedding of syndecan-1. ERK1/2 mediates the suppression of syndecan-1. The loss of syndecan-1 causes an increase in TGF-β1 secretion and expression. The limited protracted increase in TGF-β1 further enhances MMP-7 activity, leading to syndecan-1 shedding, thus forming an MMP-7/syndecan-1/TGF-β1 autocrine loop. Finally, TGF-β1 and syndecan-1 are essential for S1P-induced epithelial to mesenchymal transition. Syndecan-1 probably behaves as a brake of the autocrine loop and an operator modulated forming of the metastasis-permissive microenvironment. *P < 0.05 as compared with WT control cells (ANOVA).

S1P induced a transformed phenotype (Figure 7). S1P increased cell invasiveness (Figure 7 and 7B). S1P also altered cell morphology (Figure 7C). Cell area (Figure 7D) and aspect ratio (long-to-short axis ratio) (Figure 7E, left) were increased, and circularity (Figure 7E, right) were decreased. Interestingly, electron microscopy images showed a smoother cell surface in the presence of S1P, compared with control cells (Figure 7C). These images also showed a destroyed tether/connection between a cell and its neighboring cell in the presence of S1P (Figure 7C). This is probably positively associated with the loss of syndecan-1. Thus, S1P induced cells a phenotype shift, becoming more migratory and less adhesive. Moreover, S1P downregulated the E-cadherin marker for epithelial phenotype and upregulated the Vimentin marker for mesenchymal phenotype and transcription regulatory protein Snail. These findings, in combination, supported that S1P induced EMT. Both neutralizing TGF-β1 antibody and knockdown of syndecan-1 significantly suppressed the S1P-enhanced cell invasiveness (Figure 7A and 7B), -induced change in morphology and adhesiveness (Figure 7C–7E), - downregulated E-cadherin, and -upregulated Vimentin and Snail (Figure 7F). Taken together, syndecan-1 and TGF-β1 are essential for S1P-induced EMT.

## DISCUSSION

In the present study, we documented the relationship between sphingosine-1-phosphate (S1P) and syndecan-1, and molecular mechanism involved, as well as their roles in HCC metastasis. These works reveal a novel relationship between S1P and syndecan-1. The serum level of S1P associated with the loss of syndecan-1 in HCC. S1P activates the PI3K/AKT signaling pathways via S1P_1_, which triggers an enzyme HPSE to stimulate MMP-7 and to activate the ERK1/2 signaling pathway. MMP-7 mediated the shedding of syndecan-1, and ERK1/2 mediates the suppression of syndecan-1. The loss of syndecan-1 causes an increase in TGF-β1 production. Interestingly, the elevated TGF-β1 level is tightly regulated by the MMP-7 that mediated the shedding of syndecan-1, constituting an MMP-7/syndecan-1/TGF-β1 autocrine loop. These works also show the importance of this pathway in the HCC metastasis by demonstrating that syndecan-1 and TGF-β1 both play vital roles in the generation of a mesenchymal cell phenotype of HCC cells. These works also show that the loss of syndecan-1 with attached heparan sulfate (HS) formed a metastasis-permissive microenvironment in HCC.

S1P is a sphingolipid and is formed by phosphorylation of sphingosine [17]. S1P plays important roles in inflammatory disease and cancer through modifying the extracellular environment and regulating the cytokine actions, cell survival, growth, invasion, and neovascularization [16]. A higher level of serum S1P in tumor tissue from patients with cancer, including HCC associated with poor clinical prognosis [14, 15]. There is a family of G protein-coupled receptor responsible for S1P bound, including S1P_1-5_ receptors. The effect of S1P on cell movement is mediated via receptor-dependent pathways [24, 25]. In general, S1P_1_ is exclusively coupled with Gi protein to activate cell migration through ERK, PI3K, Akt, phospholipase C (PLC), and Rac signaling [26, 27]. In glioblastoma cells, S1P inhibited cell migration via Rho signaling in glioblastoma cells via S1P_2_ [28]. We show that, in HCC, S1P_1_ levels were elevated in HCC tumor tissues by 1.603 ± 0.792 folds, compared to surrounding NTs (Figure 2D). It is consistent with the literature [29]. Significantly, a higher S1P level associated with a higher syndecan-1 level in HCC serum, and a higher S1P_1_ level associated with a lower syndecan-1 level in HCC tumor tissues. Thus, an inverse interaction exists between S1P and syndecan-1. We demonstrate an increase in the level of S1P_2_ mRNA, which is not a significant correlation with the loss of syndecan-1. We also demonstrate that the S1P_3_ mRNA was not significant changed in HCC tumor tissues, compared with the adjacent non-tumorous. It is tantalizing to speculate that S1P induced syndecan-1 shedding and EMT in HCC via S1P_1_. This is confirmed in HCC cells by using the S1P_1_ inhibitor W146. It is worth to note that the protein levels of S1P_2_ and S1P_3_ in HCC tumor tissues were not further detected in the present study.

Syndecan-1 is a transmembrane heparan sulfate proteoglycan, which mediated the cell-matrix interaction, regulated cell adhesion and migration, and cooperated with growth factors [30, 31]. The loss or overexpression of syndecan-1 correlates with poor prognosis and aggressive phenotype in various cancers, such as breast carcinomas [32], colorectal cancer [33], prostate cancer [34], and HCC [22, 23]. We observed a loss of syndecan-1 in HCC tissue or cell, with an increase in serum or medium, implying that syndecan-1 shed from HCC into serum or medium. Higher serum levels and lower tissue levels of syndecan-1 both reflect a high tumor burden and are promising prognostic marker for HCC.

The loss of syndecan-1 in HCC cells was due to shedding and inhibition together. An S1P_1_-PI3K/AKT-HPSE-MMP-7 signaling axis mediated the S1P induced shedding of syndecan-1. An S1P_1_/HPSE-ERK1/2 signaling axis mediated the S1P suppressed synthesis of syndecan-1. Although the syndecan-1 was lost in the whole cell body, it was increased in the nucleus. The nuclear translocation of syndecan-1 might influence on syndecan-1-mediated migratory capacity [35]. We have not already evaluated the precise roles of shedding, nuclear translocation, and suppression in cell motility. Nonetheless, knockdown of syndecan-1 suppresses cell invasion and EMT phenotype. Following considering of the crucial role of syndecan-1 in cell-matrix interaction, our results imply the loss of syndecan-1 with attached HS formed a metastasis-permissive microenvironment in HCC. This study presents an obvious different role of S1P in HCC with in endothelial cells. How and why S1P has distinct roles within different cell types is still a mystery.

It had reported that TGF-β autocrine loop involved in cell growth and differentiation in transformed cells [36], and that also embroiled in the transition of human carcinoma cells into a mesenchymal phenotype [37]. We have presented here is a novel MMP-7/syndecan-1/TGF-β1 autocrine loop. The elevated TGF-β1 level is tightly regulated by MMP-7 that mediated the loss of syndecan-1. Remarkably, knockdown of syndecan-1 abolished the production of TGF-β1. Thus, production of TGF-β1 is not endless because of the substantial decrease in syndecan-1 level, implying that syndecan-1 behaves as a brake of the MMP-7/syndecan-1/TGF-β1 autocrine loop and an operator modulated forming of the metastasis-permissive microenvironment.

In conclusion, our studies characterized the interaction between S1P and syndecan-1, discovered the S1P_1_-PI3K/AKT-HPSE -MMP-7 and S1P_1_/HPSE-ERK1/2 signaling axes that responsible for shedding and inhibition of sydnecan-1, and defined an MMP-7/syndecan-1/TGF-β1 autocrine loop that plays critical roles in HCC metastasis. Those might be targeted toward the development of novel treatment strategies to reduce mortality and to improve the outcome and prognosis of patients with advanced HCC.

## MATERIALS AND METHODS

### Patient Materials

A total of 40 HCC patients (23 men and 17 women; mean age 52 ± 10 years) who underwent resection of HCC were included in the study. Pairs of HCC tumor and adjacent non-tumorous (NTs) tissues were collected, snap-frozen and kept in liquid nitrogen at −80°C. Serum samples were collected from peripheral blood and routinely stored. Control serum samples (40 samples matched to the HCC patients based on gender and age, ± 5 years) were collected from healthy subjects without any medical disease. The study was endorsed by the Ethics Committee of Sichuan University. Written informed consent was obtained from all patients and healthy individuals.

### Cell culture

Human liver hepatocellular cells (HepG2) were derived and maintained as previously described [18]. Cell lines were regularly authenticated by a genetic and morphological analysis. Noneffective scrambled (TR30021) -, HPSE (TL307138) -, MMP-7 (TL311438) -, syndecan-1 (TL309598) -shRNA plasmid (OriGene, USA) respective targeting human HPSE (NM_006665), MMP-7 (NM_002423), and syndecan-1 (NM_002997) were used to obtain the negative control (NC), HPSE-, MMP-7-, syndecan-1-stable silenced HepG2 cell lines according to the manufacturer's instructions.

### S1P stimulation and pharmacological modulators

S1P and S1P_1_ receptor selective antagonist W146 were purchased from Avanti Polar Lipid, Inc. (USA) and prepared as previously described [20, 21]. Cells were stimulated in the presence or absence of 2 μM S1P for 72 h with or without pretreatment of indicated pharmacological modulates for 30 min. Those inhibitors included: S1P_1_ inhibitor W146 (10 μM), HPSE inhibitor low molecular weight heparin (50 μg/ml), generic MMP inhibitor Ilomastat (GM6601, 10 μM, Selleck, China) and its negative control (NC, EMD Millipore, USA), PI3K/AKT inhibitor LY294002 (20 μM, Selleck, China), ERK1/2 inhibitor GDC-0994 (50 μM, Selleck, China), exogenous TGF-β1 (0.5 ng/ml, H8541, Sigma, USA), and neutralizing TGF-β1 antibody (5 μg/ml, AB-246-NA, R&D, USA).

### Measurement of S1P, serum syndecan-1, sGAG release, supernatant TGF-β1, and activities of HPSE, MMPs, and MMP-7

S1P and syndecan-1 levels in human serum were detected using S1P ELISA kit (Echelon, USA), and syndecan-1 ELISA kit (mlbio, China), respectively. The releases of sulfate glycosaminoglycan (sGAG) and the secretion of TGF-β1 in culture media were performed by using 1,9-dimethylmethylene blue (DMMB) colorimetric assay [38] and TGF-β1 ELISA kit (R&D, USA), respectively. HPSE activity in total cell lysates and cell culture supernatants was respectively quantified using HPSE ELISA kit (R&D, USA). MMPs and MMP-7 activities in culture media were determined by using Sensolyte 390 generic MMP activity kit and SensoLyte 520 MMP-7 assay kit (AnaSpec, USA), respectively.

### Quantitative real-time polymerase chain reaction (qRT-PCR)

The qRT-PCR assays were carried out as previously described [39]. The results were normalized to GAPDH and further normalized to the non-treated WT control cells. Gene expression of tested molecule was normalized to the level of glyceraldehyde-3-phosphate dehydrogenase (GAPDH) within each sample using the 2^−ΔΔCT^ methods as previously described [40, 41]. The forward and reverse primer sequences were: S1P_1_ forward, TGCGGGAAGGGAGTATGTTTG and reverse, GCAGGAAGAGGCGGAAGTTATT; S1P_2_ forward, TCATCGTCATCCTCTGTTGCG and reverse, AGAAACAGGTACATTGCCGAGTG; S1P_3_ forward, CAGTATGTTCGTGGCCCTTGG and reverse, GCCTCTT GTTGGCGTCGTAA; MMP-7 forward, GGAGAT GCTCACTTCGATGA and reverse, ATACCCAAAGAA TGGCCAAG; HPSE forward, ATTTGAATGGAC GGACTGC and reverse, GTTTCTCCTAACCAGAC CTTC; Syndecan-1 forward, TCTTTGCTGTGTGCC TGGTG and reverse, CCTCCTGTTTGGTGGGCTTC; TGF-β1 forward, AACAATTCCTGGCGATACCT and reverse, TAGTGAACCCGTTGATGTCC; GAPDH forward, CTTTGGTATCGTGGAAGGACTC and reverse, GTAGAGGCAGGGATGATGTTCT.

### Western blot analysis

Protein extraction and blotting were performed as previously described [42]. After determination of protein concentration by a Protein Determination Kit (Cayman), equal amounts (50~100 μg) of protein samples were size fractionated using SDS-PAGE, electrotransferred onto PVDF membrane (Bio-Rad) and hybridized with antibodies. Antibodies used were for S1P_1_ (TA311878, OriGene), Syndecan-1 (sc-390791, Santa Cruz), *p*-AKT (sc-33437, Santa Cruz), AKT (sc-8312, Santa Cruz), *p*-ERK1/2 (#9101, Cell Signaling), ERK1/2 (#4695, Cell Signaling), TGF-β1 (sc-52893, Santa Cruz), E-cadherin (sc-1500, Santa Cruz), Vimentin (sc-58899, Santa Cruz), Snail (#3879, Cell Signaling), and GAPDH (sc-137179, Santa Cruz). A 1:1000 dilution of those antibodies was used for detection. Densitometric quantification of bands was analyzed by the ImageJ software (version 1.50, NIH, USA).

### Immunofluorescence confocal microscopy analysis

Cells were fixed, stained, imaged, and quantified as previously described [20, 21] using LSM 510. Quantitative analyses were performed on the maximum-intensity Z-projections of Z-series stack for HS. For HS and syndecan-1 staining, mouse monoclonal antibody (1:100; 10E4 epitope, no. 370255, AMSBIO, UK) and syndecan-1 (1:100) were used, respectively.

### Invasion assays

Invasion assays were performed as previously described [43]. Cells were seeded at a density of 110^5^ cells per Transwell. After 72 h, cells were fixed in methanol and stained with crystal violet. Invaded cells were quantified in 10 microscopic fields. Results were presented as mean ± S.D of independent experiments repeated in triplicate.

### Scanning electron microscope

After treatment, cells were prepared as previously described [44]. In brief, HepG2 control and syndecan-1 shRNA cells (8 × 10^4^) were growth on the cover glass placed in 6-well plates overnight, and were treated with 2 μM S1P for 72 h with or without an addition of 5 μg/ml neutralizing TGF-β1 antibody. After fixed with 4% glutaraldehyde, the dried cell samples were gold-coated, and then images were obtained by scanning electron microscope (S-3400N, Hitachi, Japan). Changes in the cell area and morphology (including the aspect ratio and circularity) were quantified by using Image J.

### Statistical analysis

Statistical significance was determined by one-way ANOVA with either the least significant difference (LSD) test or Tamhane's *T^2^* test (depending on Levene's statistic for homogeneity of variance), except the coefficients of correlation were determined by Pearson's correlation methods, using SPSS 23 software. *P* values < 0.05 were considered statistically significant.

### Highlights

Serum S1P level in HCC positively correlated with the serum syndecan-1 level.

A significant inverse correlation existed between S1P_1_ and syndecan-1 in HCC tissues.

The loss of syndecan-1 causes an increase in TGF-β1 production.

TGF-β1 and syndecan-1 are essential for S1P-induced EMT.

S1P induced EMT via an MMP-7/Syndecan-1/TGF-β Autocrine Loop.
